# Systemic Assessment of Chronic Toxicity of Thiamethoxam on Honeybees (*Apis mellifera*)

**DOI:** 10.3390/insects16090936

**Published:** 2025-09-05

**Authors:** Meng-Jia Li, Qi-Bao He, Yi-Fan Wu, Quan Gao, A-Long Wang, Jin-Jing Xiao, Min Liao, Yong Huang, Yao-Hui Wang, Hai-Qun Cao

**Affiliations:** 1 College of Resources and Environment, Anhui Agricultural University, Hefei 230036, China; pinkmoon1231@163.com; 2 Anhui Province Key Laboratory of Crop Integrated Pest Management, School of Plant Protection, Anhui Agricultural University, Hefei 230036, China; heqibao0418@126.com (Q.-B.H.); ring191231@163.com (Y.-F.W.); quangao@ahau.edu.cn (Q.G.); alonewang163@163.com (A.-L.W.); xiaojj187012@163.com (J.-J.X.); liaomin3119@126.com (M.L.); yongh2016@163.com (Y.H.)

**Keywords:** pollinator safety, chemical pesticide, thiamethoxam, reproductive larval development, enzyme activities

## Abstract

Honeybees (*Apis mellifera ligustica* Spin.) are the most significant pollinating insects, playing a vital role in maintaining biodiversity. In healthy colonies, the reproductive success of the queen and the genetic contribution of drones are essential for long-term survival, directly affecting brood production, genetic diversity, and environmental adaptability. While the physiological and behavioral impacts of chemical pesticides on worker bees are relatively well documented, the risks to reproductive bees (queens and drones) remain poorly understood. In this study, we comprehensively assessed the effects of the widely used neonicotinoid thiamethoxam on the key developmental and physiological parameters in reproductive bees, including survival, pupation rate, eclosion rate, hormone titers, and detoxification enzyme activities. Our finding reveals that thiamethoxam exerts sublethal effects on larvae, significantly impairing the fitness of reproductive bees. Specifically, exposure altered juvenile hormone III, ecdysone titer, and acetylcholinesterase activity in reproductive larvae, with these effects showing a negative correlation with pesticide concentration. Notably, CYP450 activity exhibits a biphasic dose–response, with an initial elevation followed by a decline after reaching peak levels. These results demonstrate that thiamethoxam adversely affects the growth and development of reproductive bees, potentially compromising colony stability. By elucidating these sublethal effects, our study provides critical insights for mitigating pesticide-related threats to honeybee health. Our findings may help to scientifically and rationally avoid the potential risks of chemical pesticides to honeybees.

## Simple Summary

The unreasonable use of pesticides has contributed to a significant decrease in honeybees and unsustainable colony loss. This phenomenon has caused wide public concern in many places around the world. While the majority of current risk assessments have addressed the health of workers, they rarely refer to the queen and drone bees. Based on our previous nationwide monitoring of pesticide residues in royal jelly and other samples in the main honey-producing areas in China, we selected thiamethoxam with a high detection rate and concentration as the test pesticide to test whether it affects reproductive bee development and adversely impacts health and viability. We evaluated the effects of thiamethoxam on the entire larval development cycle of reproductive bees and conducted a comparative analysis, demonstrating that thiamethoxam significantly alters ecdysone and juvenile hormone titers in both queen and drone larvae, impairing metamorphosis and reproductive development.

## 1. Introduction

Pollination is critical to the survival and reproduction of the vast majority of plants, and terrestrial ecosystems cannot survive without pollinators [1]. Honeybees (*Apis mellifera ligustica* Spin.) are the most familiar insect pollinators and play an important role in global crop yields and biodiversity in natural ecosystems [2]. Prior to the 21st century, instances of colony collapse had already been documented in a few reports. It was not until 2007, however, that some scholars provided a formal definition of the Colony Collapse Disorder (CCD) phenomenon in a seminal publication [3]. CCD is characterized by the abrupt and widespread disappearance of worker bees from a colony, resulting in hives inhabited solely by the queen and immature brood, while honey and pollen stores remain intact and unplundered. This syndrome typically results in the eventual collapse of the colony. Declining honeybee populations and CCD are spreading globally, raising concerns about food supply shortages and diminishing biodiversity in ecosystems [4]. In recent years, beekeepers across the world have encountered unsustainably high colony losses, which resulted in the weakening of pollination in ecosystems and contributed to food supply shortages and diminishing biodiversity in ecosystems [5]. According to research findings, the reasons for honeybee decline include the fact that bee populations are exposed to multi-factorial stressors, including climate change, parasite infestations, pathogenic infections, inadequate nutrition, and pesticide exposure [6]. Due to reproductive bees not foraging, they will probably encounter more complex pesticide mixtures that accumulate in hive matrices, including wax, pollen, and honey. Thus, we estimate that they, like workers, would experience some selection for intricate stress tolerance mechanisms [7].

Environmental risk assessments are used to ensure the likelihood that a given pesticide impacts the survival of honeybee colonies [8]. However, bee larvae are also exposed to pesticides from nectar, pollen, and wax residues stored in the hive [9]. There are studies that have found that thiamethoxam exists in nectar, pollen, and plant secretions ranging from 1 to 100 mg/kg [10]. The effects of these residues likely depend on the nature of the compound and its concentration in nectar and pollen [11]. It should be mentioned that the health of the colony is indicated by the number of adult bees, while the health of the honeybee larvae determines the number of bees.

While the chronic toxicity of neonicotinoid insecticides to bees has received increasing attention in the literature [12,13,14], thiamethoxam—a major compound within this class—remains inadequately investigated with respect to its effects on reproductive bees. Research on the interaction between thiamethoxam and bees has mainly focused on worker bees [15,16], especially gatherers, but no work has been performed on drones and queens, despite their vital contribution to maintaining the stable reproduction of bee colonies. As the only male in the colony, the drone’s main task is to mate with the virgin queen to contribute half of the genetic material of the colony [17]. Because the drone does not participate in any collection activities, it cannot generate direct economic value and is usually overlooked in bee colonies. Nevertheless, given the intimate connection between the drone and the genetic framework of bee colonies, it enjoys a distinctive superiority in breeding [18]. The quality of the bee colony is determined by the reproductive drones and queens. Mated drones transfer semen, which contains half of the genetic material, to queens and enhance their fertility [19]. As a result, elements that affect drone fecundity will also have an immediate impact on the colony’s offspring. Therefore, drones could be used to optimize the characteristics of colony proliferation, productivity, and stress resistance [20].

Due to their widespread use and excellent efficacy in eliminating numerous key insect pests, neonicotinoids are considered one of the most important chemical insecticides in the world [21]. The insect nicotinic acetylcholine receptors (nAChRs), a pentameric cys-loop ligand-gated ion channel found in the insect central nervous system, are agonistically acted upon by neonicotinoids [22]. Based on total global insecticide sales, the market share of neonicotinoids has been increasing in recent years, with thiamethoxam, imidacloprid, and clothianidin particularly accounting for the vast majority [23]. Given the extensive use of neonicotinoids, environmentalists are becoming concerned about their negative effects on pollinators such as honeybees [24]. A large number of studies have shown that neonicotinoids can impact the learning and foraging of honeybees and impair colony fitness [25]. Thiamethoxam is a second-generation neonicotinoid insecticide, which is regarded as an active substance of high concern [26]. Several studies have reported that thiamethoxam impairs honeybees’ visual learning, alters decision times, and increases abnormal behaviors [27]. Existing research has demonstrated that adult drone exposure to a few pesticides negatively impacts drone fertility, but generally, little is known about how reproductive bee larvae tolerate pesticide stressors and how their stress-mitigating responses compare with each other.

Despite its regal designation, the queen does not exercise governance over the colony; rather, her primary function is reproduction, ensuring the propagation of the hive, with the vast majority of its members being her direct descendants. The drone is the sole male bee that possesses a fully developed reproductive system within the bee colony, and its healthy status and semen quality directly influence the spawning capacity and lifespan of the queen. In addition, seminal fluid is primarily derived from drone accessory glands, and it contains a complex mixture of proteins such as antioxidants, proteases, and antimicrobial proteins [28]. Several studies utilized instrumental insemination to demonstrate that drone semen initiates some post-mating changes in queens and probably plays a significant role in shaping queen quality [29]. In this work, we investigated the effects of varying dosages of thiamethoxam administered alongside fresh royal jelly, utilizing an artificial larva-rearing technique on the reproductive bees’ pupation and eclosion rates as well as its consequences on the bee larvae’s detoxification enzyme systems. Our results shed light on the widespread use of pesticides, which drastically affected both survival and physiology, exhibiting a crucial and significant generalized action that may jeopardize mating success. Thus, the colony losses observed in recent years might result from the sustained exposure of reproductive bees to multiple pesticides. We forecast that pesticide exposure may improve their defense mechanism of protection against multiple stressors but impair their ability to withstand prolonged stress, as they have few reserved resources to utilize. Future research should concentrate on the effects of multi-stressor exposures on reproductive bees and the potential genetic variability of stress resistance in the population, which is conducive to scientifically and rationally avoiding the potential risks of chemical pesticides to honeybees.

## 2. Materials and Methods

### 2.1. Breeding and Collection of Larvae

Reproductive (*Apis mellifera ligustica* Spin.) larvae were obtained from experimental apiaries kept by the Institute of the School of Plant Protection on the Anhui Agricultural University campus (Hefei, China). All colonies were kept at the same colony potential, and no pernicious substances were placed in the hives for a month before our experiments [30,31]. Adequate and healthy colonies were prepared for the experimental study. To provide healthy bee colonies, the queens were caged in the controllers, and the colonies did not contact insecticides before the experiment. Reproductive larvae were sampled from six colonies that had no Varroa infestation and or other health issues [32]. To obtain sufficient larvae, an empty spleen that spawns female and male eggs was introduced into the experimental colony and monitored for 8 h until the queen laid enough eggs [33]. The successfully hatched combs were transported to the laboratory under suitable environmental conditions for grafting.

### 2.2. In Vitro Larval Feeding 

Larvae were obtained from honeybee colonies and reared in vitro [34]. First-instar larvae (less than 24 h) were collected from the bee comb and placed in sterile 48-well cell culture plates. We used three larval diet compositions in vitro (A, B, and C) in the experiment on different days (D): diet A (D1–D3): (royal jelly/glucose/fructose/yeast extract/water = 50:6:6:1:37); diet B (D4): (royal jelly/glucose/fructose/yeast extract/water = 55:6:6:1.5:31.5); diet C (D5–D7): (royal jelly/glucose/fructose/yeast extract/water = 60:6:6:2:26). On D1–3, we fed 50 μL of diet A per larva and fed 150 μL of diet B on D4. On D5–7, each larva was fed 300 μL of diet C.

### 2.3. Chemicals and Standards

High-purity standards of thiamethoxam (purity ≥ 99.9%) were purchased from Anpel Laboratory Technologies Inc. (Shanghai, China). The total protein quantitative assay kit (A045-2) and acetylcholinesterase (A024-1) assay kit were purchased from the Nanjing Jiancheng Bioengineering Institute (Nanjing, China). Cytochrome P450 (MM-91478O2), juvenile hormone III (MM-91478O2), and ecdysone (MM-91475O2) were purchased from Jiangsu Meimian Industrial Co., Ltd. (Nanjing, China). The tested chemical was dissolved in acetone to prepare a stock solution.

### 2.4. Chronic Toxicity Assays

To expose larvae, we designed the following concentrations to measure chronic toxicity: 3 μg/L, 25 μg/L, and 2300 μg/L. The two lowest concentrations were close to the levels of residues found in nectar, pollen, and beebread [35,36,37,38,39,40,41,42]. The highest concentration was selected according to a previous study and was equivalent to 1/5 of the LC_50_ in Africanized *Apis mellifera* larvae [43]. Based on the data reported in the previous literature, we comprehensively considered and selected the low, medium, and high concentrations. On D4, we removed the unhealthy larval individuals and replaced them with healthy larvae to achieve 100% larval survival before the test. Healthy larval individuals were selected and divided into a control group and a treated group. Three replicates were set for each treatment, and each replicate selected 30 larvae. At the sixth instar (D6) for female larvae and the eighth instar (D8) for male larvae, both begin to secrete hyaluronic acid, marking the transition into the pupal stage. Larvae were carefully transferred into sterile 24-well cell culture plates (STCPs) to avoid getting injured in the process. The pupal STCPs were placed in a thermostatic incubator (35 ± 1 °C, 75 ± 5% RH, in the dark). Larval mortality needed to be recorded every day, and pupal weight and length also needed to be recorded at the red-eye stage after pupation. Pupation and eclosion rates were calculated for every treatment.

### 2.5. Assessment of Enzyme Activity and Ecdysteroid Titer

We used three detoxification enzyme activities to analyze the larvae, including acetylcholinesterase (AChE) and cytochrome P450 (CYP450). For the assessment of enzyme activity, pupal-stage bees were selected, while prepupal-stage larvae were chosen for the titer of juvenile hormone III (JH III) and ecdysone (Ecd). The experiment selected nine larvae randomly from each treatment and mixed them for the extraction. We accurately weighed the bee pupae and added 9 times the volume of physiological saline at a ratio of weight (g)/volume (mL) = 1:9. Then, mechanical homogenization was carried out under ice-water bath conditions using a handheld homogenizer. The homogenate was then centrifuged at 2500 rpm for 10 min at 4 °C in an Eppendorf (5425R) refrigerated centrifuge (Eppendorf, Hamburg, Germany). The resulting supernatant was collected for subsequent analysis. We collected the supernatant carefully and stored it at −80 °C, and then analyzed the protein concentration, ecdysteroid titer, and enzyme activities. Each sample was calculated in triplicate; a total of nine values from three biological replicates were used to evaluate parameter changes.

### 2.6. Statistical Analysis

All data are shown as mean ± standard error (SE). We used IBM SPSS Statistics 22.0 software (SPSS Inc., Chicago, IL, USA) to conduct enzyme activity and hormone titer comparisons and statistical analyses for the biological parameter assays. The survival rate data curves were tested for each compound using a Kaplan–Meier survival analysis. Larval enzyme activity, hormone titer, larval mortality, pupation rate, eclosion rate, and pupal weight and length were analyzed using a one-way analysis of variance (ANOVA), followed by Duncan’s test for multiple comparisons. Survival curves for larvae were plotted, and biological parameters and enzyme activity were assessed using the built-in methods of Origin 2019 (OriginLab Corporation, Northampton, MA, USA).

## 3. Results

### 3.1. Survival Bioassay of Bee Larvae

**Figure 1:**
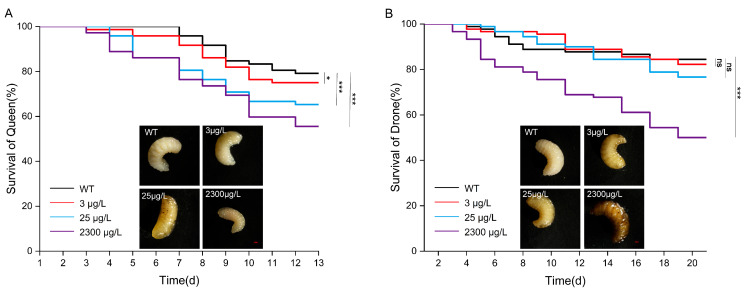
Total survival of queens (**A**) and drones (**B**) (mean ± SE) when exposed to thiamethoxam. The queen and drone larval morphology of the control group and the different treatment groups. The survival rate data curves were tested for each compound usig a Kaplan–Meier survival analysis, ns, not significant (*p* > 0.05), * *p* < 0.05, *** *p* < 0.001. Each treatment was replicated three times. Scale bar, 2 mm.

We evaluated the effects of thiamethoxam on the overall survival of queen and drone honeybee larvae in vitro and compared the chronic impacts of pesticide exposure on their development into adulthood. Relative to the control group, dietary exposure to thiamethoxam at concentrations of 3 μg/L (χ^2^ = 5.766, *p* = 0.016), 25 μg/L (χ^2^ = 65.043, *p* < 0.001), and 2300 μg/L (χ^2^ = 65.043, *p* < 0.001) significantly reduced the total survival rate of queen bee larvae (Figure 1A, *p* < 0.05). In contrast, for drone larvae, neither the 3 μg/L (χ^2^ = 0.414, *p* = 0.520) nor the 25 μg/L (χ^2^ = 0.217, *p* = 0.641) treatment produced a statistically significant effect on survival (Figure 1B, *p* < 0.05). However, exposure to 2300 μg/L (χ^2^ = 43.518, *p* < 0.001) of thiamethoxam resulted in a significant reduction in the overall survival rate of drone larvae (Figure 1B, *p* < 0.05).

### 3.2. Effects of Oral Exposure to Thiamethoxam on the Bee Pupae

**Figure 2:**
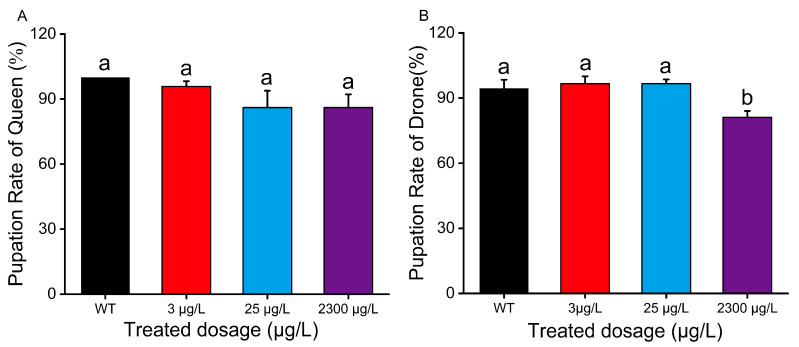
Queen pupation rate (**A**) (mean ± SE) and drone pupation rate (**B**) (mean ± SE) when exposed to thiamethoxam. Each treatment contained 30 individuals, and this was replicated three times. The pupation rate data curves were tested for each compound using a one-way analysis of variance (ANOVA), followed by Duncan’s test for multiple comparisons. Bars with different lowercase letters are significantly different at *p* < 0.05.

**Figure 3:**
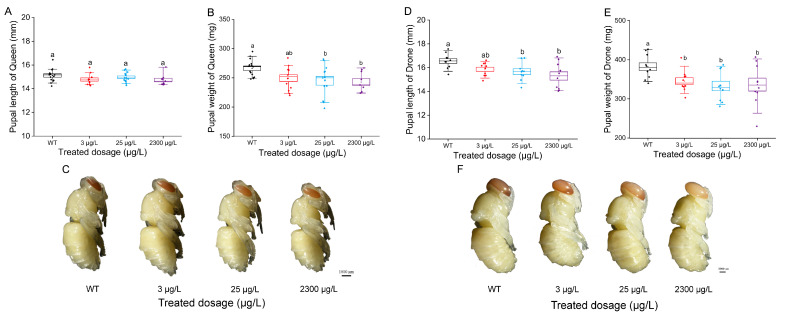
Body length (**A**) and weight (**B**) (mean  ±  SE) of the pupal stage of queen larvae reared in vitro and exposed to thiamethoxam in the diet. Body length (**D**) and weight (**E**) (mean  ±  SE) of the pupal stage of drone larvae reared in vitro and exposed to thiamethoxam in the diet. The queen (**C**) and drone (**F**) pupal-stage morphology of the control group and different treatment groups. The data curves were tested for each compound using a one-way analysis of variance (ANOVA), followed by Duncan’s test for multiple comparisons. Scale bar, 2 mm. Bars with different lowercase letters are significantly different at *p* < 0.05.

We observed and collected data on queen and drone larvae at D6 and D8, respectively, and then calculated the pupation rate. The queen pupation rates for the control group and treatment groups (3 μg/L, 25 μg/L, and 2300 μg/L) were 100%, 95.83%, 86.11%, and 86.11%, respectively (Figure 2A, *p* < 0.05), with no significant difference between the treatment groups and the control group. The drone pupation rates for the control group and treatment groups (3 μg/L, 25 μg/L, and 2300 μg/L) were 94.44%, 96.67%, 96.67%, and 81.11%, respectively (Figure 2B, *p* < 0.05). Notably, the pupation rate decreased significantly when drones were exposed to thiamethoxam at 2300 μg/L compared with other groups (Figure 2B, *p* < 0.05). Subsequently, when bee pupae reached the red-eye stage, we observed and measured their weight and length (Figure 3C,F). Exposure to thiamethoxam at the two higher concentrations (25 μg/L and 2300 μg/L) led to pronounced detrimental effects on pupal development. In queen bees, these two concentrations (25 μg/L and 2300 μg/L) significantly reduced pupal weight (Figure 3B, *p* < 0.05), further demonstrating the harmful effects of elevated thiamethoxam exposure on reproductive caste development. Specifically, drone pupae exposed to 25 μg/L and 2300 μg/L exhibited a significant decrease in pupal length compared to the control and other treatment groups (Figure 3D, *p* < 0.05), indicating that higher thiamethoxam doses may disrupt normal growth and morphogenesis during pupal development. Moreover, thiamethoxam exposure caused a notable reduction in pupal weight across multiple treatment groups. For drones, all tested concentrations (3 μg/L, 25 μg/L, and 2300 μg/L) significantly lowered pupal weight (Figure 3E, *p* < 0.05), suggesting that even low thiamethoxam concentrations could negatively affect nutrient accumulation or metabolic processes during development.

### 3.3. Effects of Oral Exposure to Thiamethoxam on the Eclosion Rate of Bees

**Figure 4:**
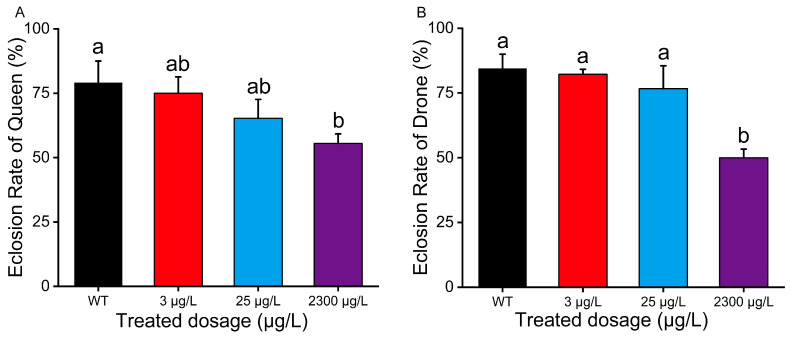
Percent queen eclosion rate (**A**) (mean ± SE) and drone eclosion rate (**B**) (mean ± SE) when exposed to thiamethoxam. Solvent treatment (acetone) served as the blank control. Each treatment was replicated three times. The eclosion rate data curves were tested for each compound using a one-way analysis of variance (ANOVA), followed by Duncan’s test for multiple comparisons. Bars with different lowercase letters are significantly different at *p* < 0.05.

When drones and queens were pupated on D-21 and D-13, respectively, we observed bee emergence and collected eclosion rate data. The queen eclosion rates for the control group and the treatment groups (3 μg/L, 25 μg/L, and 2300 μg/L) were 79.16%, 75%, 65.28%, and 55.56%, respectively (Figure 4A). The drone eclosion rates for the control group and the treatment groups (3 μg/L, 25 μg/L, and 2300 μg/L) were 84.44%, 82.22%, 76.67%, and 50.00%, respectively (Figure 4B). No significant difference existed between the control group and the 3 μg/L or 25 μg/L treatment groups, whereas both emergence and eclosion rates significantly declined in the 2300 μg/L treatment group compared to the control (Figure 4, *p* < 0.05).

### 3.4. Enzymatic Activity

**Figure 5:**
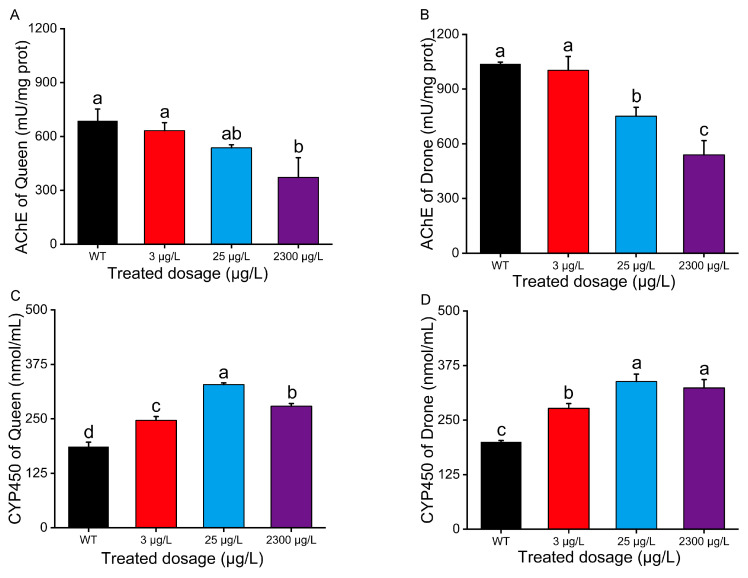
Effect of thiamethoxam on detoxification enzyme activities of queen pupa, including (**A**) acetylcholinesterase (AChE) and (**C**) cytochrome P450 (CYP450). Effect of thiamethoxam on detoxification enzyme activities of drone pupa, including (**B**) acetylcholinesterase (AChE) and (**D**) cytochrome P450 (CYP450). The data curves were tested for each compound using a one-way analysis of variance (ANOVA), followed by Duncan’s test for multiple comparisons. Bars represent the mean values ± SE of three repetitions. Bars with different lowercase letters are significantly different at *p* < 0.05.

Enzymes involved in oxidative stress and detoxification processes are recognized as sensitive biomarkers. Therefore, we examined thiamethoxam’s effect on acetylcholinesterase (AChE) and cytochrome P450 (CYP450) in reproductive bees. We assessed thiamethoxam’s impact on larval detoxification enzyme systems 72 h after a single exposure to a contaminated diet. Here, we used the whole-insect homogenates to obtain the enzyme preparations. In queen larvae, AChE activities at different thiamethoxam concentrations were 686.82 ± 65.45, 632.69 ± 43.75, 536.83 ± 16.60, and 372.41 ± 109.28 mU/mg protein, respectively (Figure 5A). For drone larvae, AChE activities were 1038.27 ± 10.01, 1002.95 ± 76.13, 751.73 ± 48.88, and 539.24 ± 78.53 mU/mg protein, respectively (Figure 5B). Although drone larvae showed higher overall enzyme activity than queens, both exhibited dose-dependent decreases. Notably, AChE activity significantly decreased in queens exposed to 2300 μg/L thiamethoxam (Figure 5A). Similarly, drone larvae showed dose-dependent AChE inhibition, with significant suppression at both 25 μg/L and 2300 μg/L (Figure 5B). CYP450 activities in drone larvae were 200.12 ± 3.14, 277.05 ± 10.96, 338.56 ± 16.92, and 323.76 ± 19.15 nmol/mL at different thiamethoxam concentrations (Figure 5D). Queen larvae showed CYP450 activities of 186.19 ± 10.17, 246.47 ± 8.86, 328.75 ± 3.82, and 279.15 ± 6.27 nmol/mL, respectively (Figure 5C). CYP450 activity displayed a biphasic response—an initial increase followed by a decline—peaking at 25 μg/L in both drone and queen bees (Figure 5C,D, *p* < 0.05).

### 3.5. Hormone Titer

**Figure 6:**
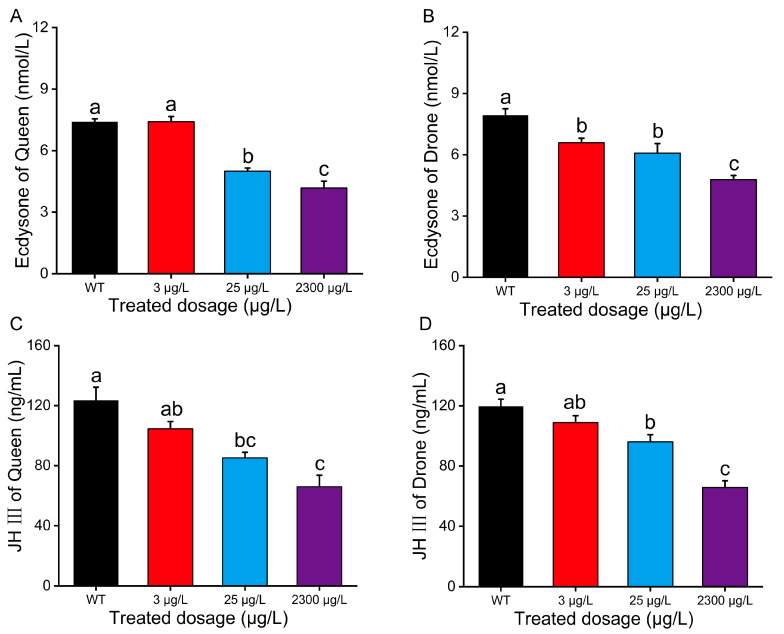
Effect of thiamethoxam on hormone titer of queen pupa, including (**A**) ecdysone (Ecd) and (**C**) juvenile hormone III (JH III). Effect of thiamethoxam on hormone titer of drone pupa, including (**B**) ecdysone (Ecd) and (**D**) juvenile hormone III (JH III). Bars represent the mean values ± SE of three repetitions. The data curves were tested for each compound using a one-way analysis of variance (ANOVA), followed by Duncan’s test for multiple comparisons. Bars with different lowercase letters are significantly different at *p* < 0.05.

Insect development and growth are primarily regulated by hormonal mechanisms, with JH III and Ecd playing crucial roles. In queen larvae, Ecd titers at different thiamethoxam exposure concentrations (3 μg/L, 25 μg/L, and 2300 μg/L) were 7.40 ± 0.15, 7.41 ± 0.25, 5.00 ± 0.15, and 4.18 ± 0.33 nmol/L, respectively (Figure 6A). For drone larvae, Ecd titers at different exposed thiamethoxam concentrations (3 μg/L, 25 μg/L, and 2300 μg/L) were 7.94 ± 0.32, 6.59 ± 0.48, 6.08 ± 1.04, and 4.79 ± 0.20 nmol/L, respectively (Figure 6B). Both reproductive bees exhibited dose-dependent Ecr suppression, with statistically significant differences (*p* < 0.05) between all treatment groups and controls, except for queen bees exposed to the lowest test concentration (Figure 6B, *p* < 0.05). JH III titers in larvae demonstrated a dose-dependent decrease following thiamethoxam exposure (Figure 6C, D, *p* < 0.05). Drone larvae exhibited JH III titers of 119.70 ± 4.74 ng/mL, 108.86 ± 4.61 ng/mL, 96.14 ± 4.69 ng/mL, and 65.74 ± 4.51 ng/mL at 3 μg/L, 25 μg/L, and 2300 μg/L, respectively (Figure 6D), Queen larvae showed corresponding values of 123.52 ± 8.74 ng/mL, 104.63 ± 4.83 ng/mL, 85.21 ± 3.70 ng/mL, and 66.02 ± 7.60 ng/mL, respectively (Figure 6C). A comparative analysis revealed significant concentration-dependent JH III suppression in both reproductive bees after thiamethoxam exposure.

## 4. Discussion

At present, honeybees have garnered increased attention as a significant pollinator, and the demand for honeybee pollination has risen along with the escalating fluctuation of crop diversification [44]. Evidence indicating a decline in the honeybee population is on the rise globally and has received extensive attention [45], while being mainly centered on worker bees with relatively little research on reproductive bees (queens and drones) [46]. A honeybee colony functions as a highly organized eusocial community that is primarily composed of sterile female workers governed by a single reproductive queen. The drone is haploid, which is produced by the parthenogenesis of an unfertilized egg cell [47]. The stabilization of a honeybee colony depends, to a certain extent, on the reproductive availability of the queen bee, which also hinges on the viability of the drone with which the queen mates [48]. Although a drone’s lifespan is short and its function is simple, the drone holds significant value in bee breeding because of its unique genetic structure [49]. The development of reproductive bees following exposure during the larval stages was the initial focus of this investigation. In this study, an artificial larval-rearing technique was employed to assess chronic oral toxicity and test the physiological index of bee larvae at different doses of thiamethoxam. The results revealed that the survival rate of bee larvae decreases gradually with the increasing dose of thiamethoxam. In the study of acute exposure, the LC_50_ value of thiamethoxam for larvae was 28.88 mg/L [43], indicating that thiamethoxam-treated African honeybee larvae reduced the survival rate, and the LC_50_ of thiamethoxam for African larvae was 14.34 mg/L of the diet (48 h) with a 95% confidence.

Based on previous studies about the acute toxicity of thiamethoxam, we hypothesized that a sublethal effect might also occur in the stage of larval development, which plays an essential role in the life cycle of a honeybee colony. Thus, the effects of thiamethoxam on the reproductive bees were investigated from the phenotypic to the physiological level following the exposure of bees, in a semi-controlled environment, from pupariation to emergence. However, despite the existence of different tested concentrations, the survival rate of drone larvae treated with a single dose of thiamethoxam was 73.50%, and that of larvae treated with double doses was 60.50% as recently observed by Gajger et al. [50]. In a study assessing the reproductive viability of honeybee queens, Ivanna V. Kozii et al. found that developmental exposure to 50 ng thiamethoxam impaired survival, with mortality rates increasing by 31–46% compared to controls. According to the study by Tavares et al. [51], the survival of larvae exposed to the low dose showed no significant difference, while those exposed to the high dose presented a significant difference. Another study demonstrated that the survival of stingless bee (*Scaptotrigona aff. Depilis*) larvae exposed to thiamethoxam was significantly impaired [52], and the group treated with the highest dose of thiamethoxam had a significant decrease in survival compared to the control group. This follows other studies, which reported that pesticide exposure may cause delayed effects during the pupal or adult stage [53,54]. Gill et al. assessed the effect of thiamethoxam and found various sublethal late effects on brownish larvae, with delays in development, and deformed adults. The reasons for chronic toxicity due to sublethal-dose thiamethoxam exposures remain unknown. This may be related to the ample time required to accumulate adequate insecticide concentrations internally to generate nerve action at central target sites, which is in keeping with the pharmacological receptor theory. This phenomenon not only explains the significance of those transitional periods in the development of reproductive bees but also expresses that those periods should be considered at risk when exposed to insecticides [51]. Previous studies by Overmyer et al. have demonstrated that thiamethoxam exerts significant sublethal effects on honeybee larval development [55]. Their experimental findings indicate that chronic exposure to environmentally relevant concentrations of thiamethoxam severely impairs larval viability and leads to a marked reduction in adult emergence rates, particularly in queen-rearing colonies. Our current findings on chronic toxicity in reproductive bees corroborate and extend these observations, revealing that elevated thiamethoxam concentrations induce statistically significant reductions in emergence success for both queen and drone. These consistent results across independent studies underscore the substantial developmental toxicity of thiamethoxam on critical reproductive castes in honeybee colonies.

The enzyme activities can be altered by pesticide exposure, which is a sensitive biomarker for assessing the physiological responses and uncovering the detoxification mechanisms of honeybees [33]. There are the main detoxification enzymes in honeybees, including AChE and CYP450, which mainly participate in the functionalization or conjugation of detoxification processes. In this study, thiamethoxam exposure significantly suppressed AChE activity in honeybee larvae. As a neonicotinoid insecticide, thiamethoxam exhibits structural homology to acetylcholine (ACh), enabling competitive binding to nicotinic acetylcholine receptors (nAChRs). This interaction induces persistent receptor activation and neuronal depolarization, which may trigger feedback inhibition of AChE synthesis or function to compensate for synaptic overstimulation. The resulting impairment of cholinergic neurotransmission likely contributes to the observed neurobehavioral deficits, including locomotor dysfunction and feeding abnormalities, ultimately reducing larval eclosion rates. These findings align with established mechanisms of neonicotinoid neurotoxicity in pollinators. Previous toxicological investigations have demonstrated that exposure to neonicotinoid insecticides imidacloprid and thiamethoxam induces significant dose-dependent modulation of CYP450 activities in honeybees when compared with field-resistant controls [56]. Quantitative enzymatic assays revealed a progressive elevation in P450 activity that exhibited a strong positive correlation with increasing insecticide concentrations, suggesting a potential detoxification response mechanism. Our analysis of CYP450 activity in pesticide-exposed reproductive bees revealed a consistent biphasic response pattern across both sexes, where enzymatic activity initially increased to maximum levels before exhibiting modest decline at higher thiamethoxam concentrations, indicating potential conservation of detoxification capacity thresholds [33,57].

Our investigation of thiamethoxam’s impact on ecdysteroid titers in reproductive bee larvae revealed significant downregulation following pesticide exposure. This might be because its sublethal dose could significantly interfere with the endocrine system of insects, thereby blocking its synthesis and signaling pathways. Transcriptomic analysis by Zhang et al. [58] demonstrated significant differential expression of ecdysone receptor (EcR) genes following neonicotinoid exposure, with thiamethoxam-resistant insect strains exhibiting markedly reduced EcR expression levels compared to susceptible counterparts. Although thiamethoxam does not directly inhibit AChE, its persistent activation of nicotinic acetylcholine receptors (nAChRs) induces neuroexcitotoxicity, which may secondarily compromise AChE function. In response, bees upregulate CYP450 expression in an effort to detoxify thiamethoxam and its metabolites. This detoxification process is energetically costly and generates oxidative stress through the production of reactive oxygen species. The resulting systemic stress disrupts endocrine homeostasis, suppressing both ecdysone and JH synthesis and signaling. JH dysregulation can accelerate behavioral maturation—such as precocious foraging—while impaired Ecd signaling disrupts development and molting processes. Collectively, these interactions form a vicious cycle: pesticide-induced stress perturbs detoxification and neuroendocrine functions, which in turn exacerbate metabolic deficits and ultimately lead to behavioral abnormalities, reduced longevity, and colony decline.

## 5. Conclusions

Our study is the first in the available literature to demonstrate the sublethal effects of the insecticide thiamethoxam on the reproductive bee larvae of a eusocial insect species. We showed that the exposure of the reproductive bee larvae to thiamethoxam may influence the survival rate, pupation rate, eclosion rate, hormone titer, and enzyme activity when present in the field. Pesticide exposure resulted in the spreading of poor-quality semen and affected the offspring, although the physical integrity of the drone was maintained. If the physiology and detoxification enzyme system of queens is strongly impacted and jeopardizes their survival, the mating success rate might be compromised and generate a shortage of healthy workers in congregation areas, which would be highly detrimental to the species. Despite being critical elements in honeybee reproduction, factors affecting reproductive bees’ survival and physiology are generally under-researched, as the overwhelming majority of available studies have focused on worker bees and the sterile female caste. This study provided a theoretical basis for the scientific and rational use of neonicotinoid pesticides and reduced the potential risks of residue to reproductive bees.

## Data Availability

The data that support the findings of this study are available in the Graphic Abstract of this article.
